# Metabolic Aspects of Anthracycline Cardiotoxicity

**DOI:** 10.1007/s11864-020-00812-1

**Published:** 2021-02-05

**Authors:** Michele Russo, Angela Della Sala, Carlo Gabriele Tocchetti, Paolo Ettore Porporato, Alessandra Ghigo

**Affiliations:** 1 Department of Molecular Biotechnology and Health Sciences, Molecular Biotechnology Center, University of Torino, Torino, Via Nizza 52, 10126 Torino, Italy; 2 Department of Translational Medical Sciences, Federico II University, Naples, Italy; 3 Interdepartmental Center of Clinical and Translational Sciences (CIRCET), Federico II University, Naples, Italy; 4 Interdepartmental Hypertension Research Center (CIRIAPA), Federico II University, Naples, Italy

**Keywords:** Cardiotoxicity, Cardiac metabolism, Doxorubicin

## Abstract

Heart failure (HF) is increasingly recognized as the major complication of chemotherapy regimens. Despite the development of modern targeted therapies such as monoclonal antibodies, doxorubicin (DOXO), one of the most cardiotoxic anticancer agents, still remains the treatment of choice for several solid and hematological tumors. The insurgence of cardiotoxicity represents the major limitation to the clinical use of this potent anticancer drug. At the molecular level, cardiac side effects of DOXO have been associated to mitochondrial dysfunction, DNA damage, impairment of iron metabolism, apoptosis, and autophagy dysregulation. On these bases, the antioxidant and iron chelator molecule, dexrazoxane, currently represents the unique FDA-approved cardioprotectant for patients treated with anthracyclines.

A less explored area of research concerns the impact of DOXO on cardiac metabolism. Recent metabolomic studies highlight the possibility that cardiac metabolic alterations may critically contribute to the development of DOXO cardiotoxicity. Among these, the impairment of oxidative phosphorylation and the persistent activation of glycolysis, which are commonly observed in response to DOXO treatment, may undermine the ability of cardiomyocytes to meet the energy demand, eventually leading to energetic failure. Moreover, increasing evidence links DOXO cardiotoxicity to imbalanced insulin signaling and to cardiac insulin resistance. Although anti-diabetic drugs, such as empagliflozin and metformin, have shown interesting cardioprotective effects in vitro and in vivo in different models of heart failure, their mechanism of action is unclear, and their use for the treatment of DOXO cardiotoxicity is still unexplored.

This review article aims at summarizing current evidence of the metabolic derangements induced by DOXO and at providing speculations on how key players of cardiac metabolism could be pharmacologically targeted to prevent or cure DOXO cardiomyopathy.

## Introduction

Doxorubicin (DOXO) is a highly effective chemotherapeutic drug belonging to non-selective class I anthracycline family [1], widely used for the treatment of several cancers, such as solid tumors, acute leukemia, lymphomas, and breast cancer [2, 3]. However, its clinical use is hampered by its cumulative and irreversible cardiotoxicity, which leads to myocardial dysfunction manifesting as aberrant arrhythmias, ventricular dysfunction, and congestive heart failure, even years after chemotherapy cessation [4–6].

As the number of cancer survivors is steadily increasing, the long-term side effects of DOXO administration are becoming ever more apparent [7]. Despite the exponential growth of the field of cardio-oncology in the last decade, the molecular mechanisms underlying DOXO-induced cardiotoxicity have not been fully elucidated yet [8]. The finding that antioxidants fail to prevent DOXO-induced cardiotoxicity has challenged the classical view according to which oxidative stress is the main determinant of the cardiac side effects of DOXO, suggesting the involvement of additional mechanisms [8, 9]. Among the theories that have been proposed are mitochondrial dysfunction [10], DNA damage [11], defects in iron handling [10], apoptosis [12], and dysregulation of autophagy [13–15].

Although the exact mechanism of DOXO cardiotoxicity remains to be defined, mitochondrial damage and accumulation of dysfunctional mitochondria have been shown as key hallmarks of DOXO-induced cardiotoxic effects [13]. Mitochondria constitute around 50% of the cardiomyocyte volume and are vitally important for energy generation. As DOXO accumulates in the inner mitochondrial membrane by binding cardiolipin, this perturbs mitochondrial protein function and uncouples mitochondrial respiratory chain complexes, eventually impairing ATP production [16]. Moreover, the ATP deficiency linked to DOXO cardiotoxicity has been directly correlated to alterations of mitochondrial energy metabolism and bioenergetics.

The myocardium can fulfill the elevated metabolic requests thanks to an incredible metabolic flexibility according to which ATP can be generated starting from a variety of energy substrates such as glucose, fatty acids, and ketone bodies. Of note, build-up of each of these carbon sources is associated with increased rates of cardiovascular diseases [17], and, in general, metabolic dysregulations play a critical role in the pathophysiology of heart failure (HF) [18, 19].

The association between metabolic dysregulation and cardiotoxicity has been demonstrated with different cancer therapies, such as copanlisib in relapsed follicular lymphoma [20], nilotinib in chronic myelogenous leukemia [21, 22], and androgen deprivation (AD) in prostate cancer [17], which were found associated to glucose dysregulation and hyperglycemia, or increased cholesterol level. Multiple studies have shown that AD therapy consistently increase insulin resistance, total cholesterol, and the rate of incident diabetes mellitus leading to increased risk of myocardial infarction and sudden cardiac death [23, 24]. However, less is known about the cardiac metabolic dysregulations involved in DOXO cardiotoxicity. Important clues come from a recent clinical study conducted in breast cancer patients treated with anthracyclines [25•], where a metabolite profiling approach has been used to define the early metabolic changes associated with the development of cardiotoxicity. Patients who developed cardiotoxicity display changes in citric acid and aconitic acid, along with an increased level of purine and pyrimidine metabolites in the plasma, that may be related to the systemic DNA damage induced by chemotherapy [25]. Of note, the identification of early metabolic changes as well as the measurement of circulating metabolites in the plasma could provide insight into the mechanisms associated with the development of DOXO cardiotoxicity.

In further support of the importance of exploring metabolic changes linked to DOXO treatment, there is growing evidence that drugs approved for the treatment of metabolic diseases, such as diabetes, could protect against anthracycline cardiotoxicity. Among them, two anti-diabetic agents, metformin (MET) and empagliflozin (EMPA), have shown promising results since, along with their glucose-lowering effects, they protect against the development of cardiometabolic diseases as well as DOXO-related cardiotoxicity [26, 27]. Moreover, empagliflozin, a SGLT2 inhibitor, exhibits protective effects in DOXO-induced HF in mice without diabetes [27•]. Taken together, these findings suggest that an improved understanding of the mechanisms underlying the regulation of cardiac metabolism in response to DOXO treatment may lead to the identification of novel pharmacological targets as well as the development of new strategies to prevent the cardiotoxic effects of DOXO in cancer patients.

Here, we focus on the description of the molecular processes governing cardiac metabolism whose deregulation has been linked to DOXO cardiotoxicity. Moreover, we discuss how the identification of key players of cardiac metabolism may be instrumental to improve and refine current therapeutic strategies.

## DOXO cardiotoxicity and iron metabolism

**Fig. 1:**
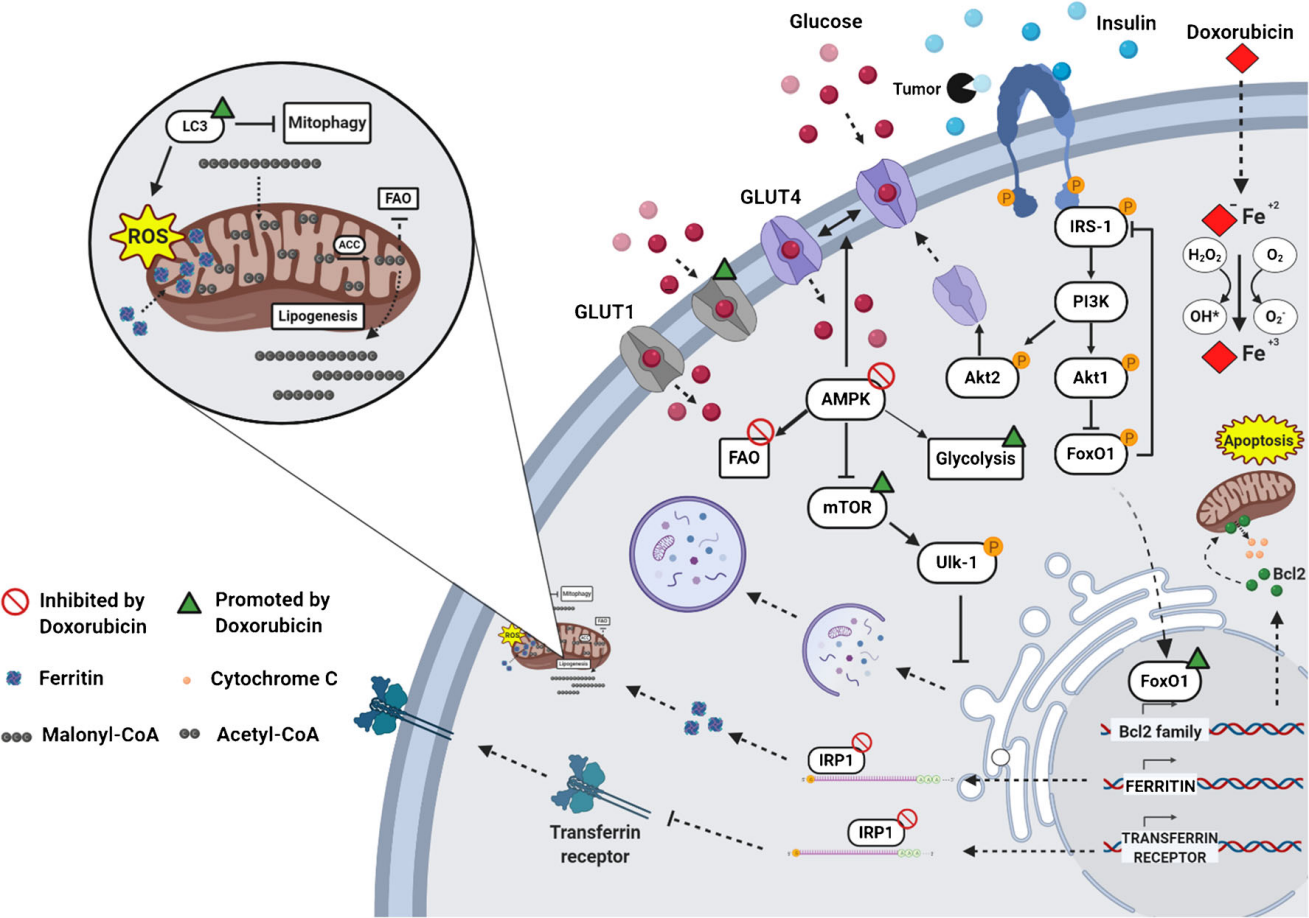
Metabolic changes induced by DOXO in cardiomyocytes. DOXO interferes with Fe^2+^ metabolism, leading to activation of ferroptosis through ROS production, disruption of IRP-1 activity, and iron accumulation into mitochondria. These events are hallmarks of mitochondrial dysfunction that leads to a block of fatty acid oxidation (FAO) and an increase in glycolysis, as a consequence of AMPK inhibition. Acetyl-CoA carboxylase (ACC), a direct downstream target inhibited by AMPK, is overactivated and catalyzes the formation of Malonyl-CoA, blocking FAO irreversibly. At the plasma membrane, DOXO promotes glucose uptake via GLUT4 through insulin-mediated activation of AMPK and AKT2. In addition, DOXO increases the expression of GLUT1, an insulin-independent glucose transporter, normally absent in the adult heart. Following the insulin desensitization induced by tumor-secreted factors, AKT1 signaling is disrupted and promotes FOXO1 nuclear translocation, inducing the activation of the apoptotic pathway through the expression of pro-apoptotic members of the Bcl-2 family. Finally, DOXO cardiotoxicity has been linked to autophagy dysregulation. DOXO inhibits autophagy by activating mTOR or by blocking AMPK, resulting in accumulation of undegraded autophagosomes and mitochondrial dysfunction with increased production of ROS. This figure was created with BioRender.com*.*

Impairment of cellular iron metabolism has been suggested as a main source of reactive oxygen species (ROS) in DOXO-induced cardiotoxicity, a theory referred to as “ROS and iron hypothesis” [28, 29]. It has been demonstrated that inside the cell DOXO is reduced to a cytotoxic semiquinone radical (SQ) that is rapidly converted back to the original molecule using O_2_ as an electron acceptor [30, 31]. This process leads to superoxide formation that is detoxicated in H_2_O_2_, either spontaneously or by superoxide dismutase activity (Fig.1). The cellular pool of chelatable and redox-active iron, defined as labile iron pool (LIP), strongly reacts with H_2_O_2_, generating ROS through Fenton reaction. Furthermore, LIP can directly interact with DOXO, creating DOXO-Fe complexes that drive ROS production [32, 33]. In support of this evidence, it is reported that DOXO interferes with mechanisms involved in cellular iron homeostasis. First, DOXO modulates the mRNA maturation of transferrin receptor and ferritin, through irreversible inactivation of the RNA-binding activity of iron regulatory proteins 1 and 2 (IRP-1 and 2) (Fig. 1) [34, 35]. Moreover, DOXO disrupts the cellular localization of iron, increasing iron/ferritin binding in the cytosol [36] and reducing its release from cellular storages, such as mitochondria (Fig. 1) [35]. In agreement, a mouse model of hereditary hemochromatosis (HH), in which the lack of the *Hfe* gene drives an aberrant iron accumulation in the heart and other organs, is characterized by increased iron accumulation into mitochondria and high susceptibility to DOXO cardiotoxicity. Thus, in response to DOXO treatment, the cytosolic iron concentration is maintained at physiological levels through reduced mobilization of cellular storages and ferritin turnover, but its accumulation within mitochondria compromises mitochondrial iron metabolism [10]. Ichikawa et al. demonstrated, both in vitro and in vivo, that overexpression of the mitochondrial transporter ABCB8 facilitates the efflux of iron from mitochondria, reduces ROS production, and protects against DOXO-induced cardiotoxicity [10]. Iron accumulation into mitochondria has been linked to ferroptosis, a recently described form of iron-dependent cell death, which is morphologically, biochemically, and genetically distinct from apoptosis, necrosis, and autophagy. Ferroptosis is featured by mitochondria iron accumulation and lipid peroxidation [37] and has been previously associated with other pathologies, such as cancer [38], stroke [39], and ischemia/reperfusion injuries [40]. Fang and colleagues revealed for the first time the role of ferroptosis in DOXO-induced cardiomyopathy. Mice defective for canonical activators of necroptosis or apoptosis or both, Ripk3 −/−, Mlkl −/−, or Fadd −/−Mlkl −/− respectively, showed typical hallmarks of ferroptosis in cardiomyocytes after DOXO administration. This study demonstrates that ferroptosis is triggered by heme oxygenase-1-mediated heme degradation through an Nrf2-dependent mechanism that drastically induces iron overload into mitochondria and ferroptosis activation [41].

Strategies to reduce iron accumulation into mitochondria in response to DOXO, using, for example, iron chelators, have been explored. Dexrazoxane is the unique molecule approved by FDA for the treatment of DOXO cardiotoxicity, for its dual activity as inhibitor of topoisomerase 2β (Top-2β) [42] and iron chelator [41]. By limiting mitochondria iron accumulation in cardiomyocytes [41•], dexrazoxane prevents the activation of apoptotic and ferroptotic pathways. Nevertheless, several side effects have been linked to the use of dexrazoxane, including the development of secondary malignancies, myelosuppression [43], and reduction of DOXO antitumoral efficacy as a consequence of the inhibition of the topoisomerase 2 isoform expressed in cancer cells, Top-2α [44, 45]. Nowadays, some of dexrazoxane-associated side effects have been retracted [46, 47], and further studies elucidate that the cardioprotective effect of dexrazoxane is mainly linked to its inhibition of Top-2β than its iron-chelating property [48].

On the other hand, specific iron chelators, such as deferiprone [10], deferoxamine [49], and deferasirox [50], failed to counteract DOXO-mediated cardiotoxicity, probably due to their limited lipophilic properties and accessibility to iron mitochondrial storage [51]. Instead, a mild protection against DOXO toxicity has been documented with the small lipophilic iron chelator pyridoxal isonicotinoyl hydrazone and its analogue [52]. Interestingly, the ferroptosis inhibitor ferrostatin-1 has been proved to reduce iron-mediated lipid peroxidation [53, 54]. Mice treated with ferrostatin-1 are protected against DOXO-induced cardiotoxicity, suggesting the use of this molecule as a valid alternative to dexrazoxane [41]. Overall, this evidence suggests that specific iron chelator molecules fail to show a significant cardioprotective effect, likely because of their inability to reach iron storage into mitochondria. In this scenario, the inhibition of ferroptosis may represent a new promising approach to target one of the multiple mechanisms driving DOXO cardiotoxicity.

## Cardiac metabolic changes triggered by DOXO: a focus on fatty acid oxidation and glycolysis

Cardiac metabolism is a highly sophisticated mechanism that in physiological conditions uses fatty acids (FAs) as a major source for catabolic reactions while switching to glycolysis in response to several pathological insults [18]. Despite the glycolytic switch that represents an early compensatory event, persistent glucose usage eventually turns maladaptive and leads to energetic failure, where glycolysis and impaired mitochondrial function do not allow cardiomyocytes to meet the cellular energy demand [55]. Studies with animal models have shown that cardiac insulin resistance and metabolic modifications, such as reduced mitochondrial oxidation of glucose, lactate, and fatty acids, are putative early markers of heart stress [56]. In agreement, the inhibition of glucose uptake consequent to insulin signaling desensitization has been identified as one of the prevalent risk factors for HF, and disruption of physiological cardiac metabolism adaptation has been associated with worse prognosis [57]. Despite these observations, the use of insulin-sensitizing agents failed to show improvements in patients and, on the contrary, has been associated with potential risk of cardiac side effects [58]. In line with this evidence, Taegtmeyer and co-workers have proposed insulin resistance as a physiological adaptation to non-ischemic heart damage, protecting cardiomyocytes from substrate overload in dysregulated metabolic states [59]. Impairment of insulin signaling has been reported to reduce glucose uptake and activate fatty acid oxidation in an AMPK-dependent manner [60].

Similarly, cardiotoxic chemotherapeutic drugs have been shown to impair intracellular mechanisms controlling cardiac metabolism [61•]. Specifically, DOXO induces the systemic insulin resistance typical of type II diabetes, with augmented serum triglyceride and blood glucose levels [62, 63], and, at the same time, triggers massive cardiac glucose uptake [64, 65]. Furthermore, DOXO has been demonstrated to affect gene expression involved in aerobic fatty acid oxidation and anaerobic glycolysis (Fig. 1) [66].

A central role in this process is exerted by AMPK, the master sensor of cellular energy status, that acts as a “fuel gauge” [67]. AMPK triggers long-term catabolic pathways that generate ATP, including fatty acid oxidation and glycolysis, while downregulating processes that are dispensable for short-term cell survival, such as the biosynthetic metabolism that rapidly consumes the ATP pool [68]. DOXO-mediated disruption of AMPK drives metabolic disarrangements and cellular substrate overload [69]. Experimental evidence shows that DOXO-induced AMPK inhibition increases glucose uptake after 2 weeks of treatment [70], probably due to concomitant expression of GLUT1 [71], an insulin-independent glucose transporter normally absent in the adult heart. Furthermore, Malonyl-CoA overproduction by acetyl coenzyme A carboxylase (ACC), an enzyme directly inhibited by AMPK [72], irreversibly blocks FAO and increases lipid synthesis and accumulation (Fig. 1) [73]. In agreement, cardiomyocyte-specific overexpression of adipose triglyceride lipase limits FA accumulation and shows a beneficial effect on cardiac function after DOXO treatment [74].

Additionally, in response to cellular stress, AMPK inhibits the activity of enzymes that reduce and consume ATP, such as creatine kinase [75]. DOXO impairs the high-energy phosphate pool through direct inhibition of AMPK [72] and creatine kinase (CK) system [76], reducing the phosphocreatine-to-creatine (PCr/Cr), PCr-to-ATP (PCr/ATP), and ATP-to-ADP (ATP/ADP) ratios [77]. In line with these observations, the recovery of AMPK activity exerts beneficial effect on mitochondria, reducing oxidative stress and preserving mitochondrial energy production [78].

The pivotal role of the AMPK pathway in the cardiac metabolic rearrangements induced by DOXO has been recently confirmed in cardiomyocytes derived from human-induced pluripotent stem cells (hi-PSCs), which have been established as a powerful model for drug toxicity screening on cells isolated from cancer patients under chemotherapy regimen [79••]. In these cells, impairment of gene modulating cardiac metabolism is one of the main effects of chemotherapeutic agents, including DOXO [80]. The use of specific AMPK-inducing agents was proven effective in counteracting the bioenergetic failure linked to the use of trastuzumab [80] and might be a new strategy to counteract the development of cardiotoxicity during chemotherapy regimens. Among these AMPK-restoring agents is metformin, a hypoglycemic drug used to treat patients with type 2 diabetes, which is known to trigger the AMPK pathway in insulin-sensitive organs, such as the heart [81]. Notably, several studies have reported the cardioprotective effects of metformin against DOXO-induced toxicity [26, 82, 83]. Furthermore, metformin also displays an AMPK-dependent antitumoral activity [84], which makes this molecule a new promising agent to treat patients that suffer both HF and cancer.

Importantly, cancer and cardiovascular diseases are known to share several risk factors, including aging, smoking, overweight, and physical inactivity, but whether these two disease conditions are directly linked is still to be defined [48, 85]. In this context, metabolic diseases have emerged as a common risk factor for both cancer and heart failure [86–89]. Moreover, a clinical study has reported that patients with comorbidities, such as diabetes, dyslipidemia, and obesity, exhibit higher incidence of DOXO-related cardiotoxicity [9]. All these indications suggest that metabolic diseases affect the clinical outcome of patients subjected to DOXO treatment. In this context, insulin signaling plays a fundamental role, in modulating both heart metabolism and cancer growth, with AMPK being one of the main regulators.

In the following paragraph, we will describe how advanced cancer itself dramatically interferes with the cardiac insulin pathway further exacerbating drug-induced toxicity.

## Insulin resistance at the crossroad of tumor growth and DOXO cardiotoxicity

Metabolic diseases, such as obesity and diabetes, significantly increase the incidence of HF in patients, where insulin resistance is a common risk factor. Insulin desensitization occurring in this state drastically reduces the important effects of insulin on cardiac tissue. Insulin receptor is widely expressed on the surface of many cell types, including cardiomyocytes, where upon ligand binding and insulin receptor substrates (IRS) 1 and 2 are recruited. IRS1 more than IRS2 is fundamental for regulation of the PI3K/Akt pathway and the MAP kinase cascade, such as ERK, both involved in the control of metabolism and cell survival [90]. Three members of the AKT family are known, AKT1, AKT2, and AKT3, but how these isoforms differentially contribute to cardiac cell function is not completely clear. It has been established that AKT1 is required for cardiomyocyte survival, while AKT2 is essential for the modulation of genes involved in cardiac metabolism. Indeed, AKT2 promotes glucose uptake through the mobilization and fusion of GLUT4-containing vesicles to the plasma membrane (Fig. 1) [90]. Despite the role of AKT during cardiac stress condition is still debated, it is reported that short-term AKT activation may exert cardioprotective effects, enhancing glycolysis and reducing oxidative phosphorylation. Controversially, chronic and long-term activity of AKT1 in the adult heart is associated with high risk of cardiac complications and reduced mitochondrial functions. Following insulin stimulation, AKT1 phosphorylates and blocks FOXO1 nuclear translocation, inhibiting the expression of pro-apoptotic proteins belonging to the Bcl-2 family (Fig. 1) [91]. FOXO1 has emerged as one of the key players in chronic metabolic diseases, promoting hyperglycemia and glucose intolerance [92]. In physiological conditions, pro-survival stimuli was induced by insulin repress FOXO1 activity through PI3K/AKT1 pathway. Following stress stimuli, FOXO1 translocates in the nucleus and induces a negative feedback on insulin pathway through a JNK-dependent mechanism that drastically reduces IRS-1 activity (Fig.1) [93].

Although the imbalance of insulin signaling has been extensively studied in several models of obesity and type 2 diabetes, only a few studies have addressed its role in DOXO-induced cardiotoxicity, and the underlying molecular mechanisms are still poorly understood. Recent studies demonstrate that aberrant FOXO1 activity is responsible of DOXO-induced cardiotoxicity and its specific pharmacological targeting has been shown to ameliorate the cardiac outcome [94, 95].

In addition to chemotherapy, the tumor itself can negatively affect cardiac insulin signaling. Interestingly, Thackeray et al. have reported that advanced cancer contributes to the impairment of cardiac insulin signaling through secretion of insulin-degrading enzymes, massive glucose adsorption, and reduced production of pancreatic insulin. In this scenario, other cancer-mediated mechanisms, such as promotion of proteolysis by ubiquitin-proteasome and autophagy-related lysosomal pathways, mitochondrial dysfunction, impairment of catabolism and anabolism reactions, and release of the proinflammatory cytokines such as IL-6 and TNF-α [96, 97], further contribute to increasing the risk of heart failure development [91]. In agreement with the well-established pro-survival role of insulin-stimulated AKT1 pathway in cardiomyocytes, administration of low-dose insulin rescues cardiac function in tumor-bearing mice by restoring AKT signaling and autophagy inhibition in cardiomyocytes, without affecting cancer glucose uptake [98••]. Furthermore, expression of a constitutively active form of AKT1 by adenoviral vector prevents heart damage and protects mice from DOXO-induced cardiotoxicity [99], suggesting that the lack of insulin-mediated AKT1 activation during cancer progression could aggravate the cardiotoxicity induced by DOXO. In agreement, previous report shows that insulin depletion is associated with increased accumulation of DOXO into the heart and reduced cardiac function [100].

In addition to defective insulin signaling, the massive glucose uptake by the tumor can deprive cardiac cells of a pivotal energetic source during stress conditions [98]. Particularly, as described by Warburg in 1920, malignant cells based their energy production on the use of glycolysis and generate lactate. This metabolic adaptation, called “Warburg effect”, confers the ability to cancer cells to survive in several stress conditions, including anaerobic environment of solid tumor inner mass. In this scenario, the use of 2-deoxyglucose (2-DG), a glucose analogue which blocks glycolysis, represent an interesting therapeutic strategy to treat cancer. 2-DG is phosphorylated to 2-DG-6-P inside the cell by hexokinase and cannot be further metabolized. It is thought that 2-DG-6-P competes with glucose utilization into glycolysis and drastically reduces energy production of cancer cells. Moreover, despite that 2-fluorodeoxy-D-glucose is a more potent glycolysis inhibitor, the main effect of 2-DG is the inhibition of N-linked glycosylation process, causing its high structural similarity to Mannose. The block of oligosaccharide formation required for the assembling of structural lipids and maturation of glycoproteins has been observed to induce tumor cells’ death even in aerobic condition [101, 102]). Moreover, further studies were conducted to investigate the combining of 2-DG with others antineoplastic agents. In vivo evidence established that 2-DG co-treatment with adriamycin or paclitaxel increased their antitumoral efficacy against osteosarcoma and non-small cell lung cancers [103]. Previous work showed that caloric restriction treatment based on the administration of 2-DG prevents DOXO-mediated cardiotoxicity through several mechanisms, including activation of AMPK-dependent mechanism [104].

Of note, the targeted therapy with a 2-DG-based adriamycin complex showed promising results, by specifically targeting tumor growth and, at the same time, limiting the organ toxicity of anthracyclines in vivo [105]. Overall, these findings suggest that the tumor itself negatively impacts on cardiac function through secreted factors that act in an endocrine manner and identify dysregulation of the cardiac insulin pathway as a major mechanism whereby the tumor negatively affects cardiac cell survival (Fig. 1).

## Autophagy at the crossroad of metabolism and cell survival in DOXO cardiotoxicity

Autophagy is a highly conserved process which is aimed to maintain cell and tissue homeostasis, promoting the elimination of damaged and long-lived organelles and misfolded proteins under both physiological and pathological conditions [106, 13]. Importantly, autophagy plays an essential role in the regulation of cellular metabolism, both in normal conditions and in the setting of energy depletion, since it has been involved in the regulation and mobilization of energy stores, such as lipids and glycogen [107]. Accumulating evidence indicates that the cardiac side effects of DOXO may be closely related to a dysregulation of autophagy signaling and an imbalance in cellular metabolism, leading to intracellular Ca^2+^ accumulation, energy depletion, and mitochondrial dysfunction [108]. However, there is still controversy on whether DOXO inhibits or activates autophagy and whether autophagy activation has a beneficial or maladaptive role in this process [14].

Several studies have revealed that DOXO interferes with the initiation of the autophagic process by modulating the two main regulatory pathways [109], AMPK and mammalian target of rapamycin (mTOR). AMPK and mTOR promote and inhibit autophagy, respectively, by finely regulating the activity of the autophagy activating kinase Ulk-1 (Fig. 1). AMPK is the main metabolic sensor of the cell and is sensitive to changes in AMP:ATP ratio that is indicative of the cellular energy state. In low energy state, activation of AMPK relieves mTOR-inhibition of ULK1, leading to induction of autophagy [110]. Conversely, in the presence of high levels of energy substrates, AMPK activity is antagonized by mTOR which inhibits autophagy [111].

It has been shown that cardiac AMPK is inhibited in response to DOXO [72, 112]. Although the exact mechanism of such regulation remains elusive, r-activation of AMPK has been proposed as a therapeutic strategy to counteract DOXO-induced HF, and the cardioprotective effects of this approach have been linked to reactivation of autophagy [113]. Importantly, promoting a negative energy balance before DOXO treatment, i.e., via starvation or exercise, restores AMPK signaling and autophagy and ultimately reduces DOXO-induced cardiotoxicity [114]. For instance, dietary restriction in rats treated with DOXO modulates the ATP:AMP ratio inducing AMPK activation, increasing fatty acid oxidation rates and ATP levels, and ultimately leads to improved cardiac function [115]. In addition, AMPK activation, and the ensuing reduction in apoptosis and increase in autophagy, was further achieved in DOX-treated rat neonatal cardiomyocytes with the caloric restriction mimetic 2-deoxyglucose [104].

### Mitochondrial dysfunction at the interplay of autophagy and metabolism

The exact link between autophagy and metabolism regulation in the pathogenic sequelae of DOXO cardiomyopathy is still to be defined. However, the prevailing view is that DOXO-induced mitochondrial dysfunction and the ensuing production of reactive oxygen species stand at the crossroad of these two cellular processes. As a consequence of its accumulation within mitochondria, DOXO uncouples mitochondrial respiratory chain complexes, eventually impairing ATP production [16]. In keeping with this model, cardiomyocytes exposed to DOXO exhibit low levels of ATP associated with dysregulation of autophagy [116]. Thus, DOXO cardiotoxicity directly contributes to ATP deficiency, altering mitochondrial energy metabolism and bioenergetics [117], even though it is still debated whether ATP deficiency is the trigger or the result of autophagy deregulation.

Compelling evidence reveals that mitochondrial autophagy or mitophagy is defective in models of DOXO-induced cardiotoxicity [118]. DOXO disrupts cardiac mitochondrial autophagy by inhibiting lysosomal biogenesis and fusion with autophagosomes, thus preventing proper digestion of damaged mitochondria engulfed by autophagosomes [119, 120]. Recently, a comprehensive study by Abdullah et al. showed a direct association between autophagy dysregulation and defects in mitochondrial respiration in the development of DOXO-associated cardiomyopathy [118••]. In this study, both in vivo and in vitro analyses showed that DOXO cardiotoxicity results in a gradual accumulation of autophagosomes (Fig. 1); DOXO-induced autophagosome accumulation, in turn, results in altered expression of proteins involved in the regulation of mitochondrial dynamics and oxidative phosphorylation (OXPHOS and PDH proteins) and in mitochondrial respiratory dysfunction [118••]. Mitochondria isolated from both DOXO-treated hearts and intact neonatal cardiomyocytes exposed to DOXO show decreased oxygen consumption rate, indicating a suppression of mitochondrial bioenergetics [118••]. Such mitochondrial dysfunction could result from defects in mitochondrial substrate uptake or in the activity of the entire TCA cycle, causing cardiomyocyte death by ATP deprivation.

In agreement, another study reports that DOXO-treated cardiomyocytes exhibit decreased levels of ATP which, in turn, activate autophagy [121]. This study demonstrates that DOXO induces the production of 4-hydroxynonenal (4-HNE), a product of lipid peroxidation which is toxic to the heart and that mediates autophagy activation through lipid peroxidation-derived aldehydes [121]. On the other hand, DOXO reduces the expression of the mitochondrial aldehyde dehydrogenase (ALDH2) [122], which has been shown to mediate cardioprotective effects by reducing the production of 4-HNE and ROS [123, 124]. ALDH2 controls 4-HNE-induced autophagy via the regulation of AMPK-Akt-mTOR-signaling pathway. The ALDH2 activator Alda-1 was shown to prevent DOXO effects in neonatal cardiomyocytes, such as downregulation of Akt phosphorylation and upregulation of autophagy proteins like Beclin-1, Atg5, and LC3-II [121]. In further support of a link between ALDH2 and autophagy regulation in response to DOXO, the autophagy inducer rapamycin could abolish the protective action of Alda-1 against DOXO-induced cardiomyocyte dysfunction, whereas the autophagy inhibitor 3-MA reduced DOXO cardiotoxicity [121]. A similar study by Ge et al. demonstrated that ALDH2 knock-in mice treated with DOXO had better cardiac function compared to DOXO-treated wild-type mice [125]. Taken together, these results suggest that promoting ALDH2 expression and inhibition of 4-HNE-induced autophagy may be a plausible approach to reduce DOXO-induced cardiac dysfunction.

Another possible link between mitochondrial metabolism dysfunction and autophagy dysregulation in DOXO-induced cardiotoxicity could be represented by intracellular calcium signaling [126, 127]. Decuypere et al. reported intracellular Ca^2+^ as one of the regulators of autophagy [128]. In healthy conditions, intracellular Ca^2+^ signaling suppresses autophagy, while under stress conditions and low energy production Ca^2+^ signaling is enhanced and stimulates autophagy. It has been reported that DOXO perturbs the expression of Ca^2+^-handling proteins and alters Ca^2+^ homeostasis, causing mitochondrial dysfunction and apoptosis in the myocardium [126]. By disrupting Ca^2+^ handling, DOXO dysregulates autophagy in human cardiac progenitor cells (hCPCs), which are important regulators of myocardial homeostasis [127]. In hCPCs, the cytotoxic effects of DOXO induce abnormal cytosolic Ca^2+^ accumulation which, in turn, disrupts mTOR-mediated regulation of autophagy. Additionally, DOXO reduces the expression of the autophagosome marker LC3 and of an anti-senescence marker, SMP30, leading to reduced autophagosome formation and cellular viability, respectively [127]. Accordingly, autophagy activation with the mTOR inhibitor rapamycin rescues DOXO cardiotoxicity in hCPCs, with a significant reduction in DOXO-mediated cytosolic Ca^2+^ accumulation and restored autophagosome formation as well as SMP30 expression [127].

Rapamycin has been also shown to alleviate the autophagic interruption mediated by insulin-like growth factor II receptor α (IGF-IIRα) in DOXO-treated H9c2 cells [129]. IGF-IIRα is a novel stress-inducible contributor to cardiac damage which has been linked to DOXO-induced oxidative stress and autophagy alteration [129]. Interestingly, IGF-IIRα overexpression in combination with DOXO treatment increases LC3 expression and perturbs autophagosomal formation, impairing autophagy both in vitro in H9c2 cells and in vivo in transgenic rat models [129].

Overall, these findings suggest that DOXO-mediated dysregulation of autophagy drives mitochondrial dysfunction via different cytosolic and mitochondrial signaling axes and that restoring autophagy may be a valuable therapeutic approach to target DOXO toxicity.

### Metabolic agents as potential strategies to restore autophagy in DOXO cardiotoxicity

Currently, there are no specific treatments for DOXO cardiotoxicity, and cancer patients experiencing cardiac issues are primarily treated with standard heart failure medications, such as renin angiotensin system blockers and beta blockers. As discussed above, reactivation of AMPK has been proposed as a therapeutic option to treat heart failure associated with different metabolic diseases. Intriguingly, the anti-diabetic drug and AMPK activator, metformin, has been shown to improve cardiac function in a diabetic OVE26 mouse model by increasing autophagy activity [130]. Consistent with these findings, Zilinyi and co-workers reported that co-administration of DOXO and metformin increases autophagic activity and confers cardioprotection in a rat model [26]. This study shows that metformin restores LC3 levels and induces AMPK autophagy initiation, leading to improved cardiac function and reduced DOXO cardiotoxicity [26].

Recently, new hypoglycemic drugs like SGLT2 inhibitors have been shown to restore DOXO-mediated dysregulation of autophagy and to improve cardiac function [131, 132•]. Among these, empagliflozin (EMPA) has showed important cardioprotective effects in both diabetic and non-diabetic in vivo models undergoing DOXO treatment [27]. Previous work with diabetic animal models treated with EMPA has led to the hypothesis that EMPA prevents heart failure by improving ATP generation and thereby enhancing cardiac efficiency [132•, 133]. Consistently, Zucker diabetic fatty rats treated with EMPA show enhanced cardiac autophagy via increased AMPK activation [132•]. Moreover, EMPA enhances the cardiac energy pool by increasing cardiac energy production from glucose and fatty acid oxidation, whereas it reduces the cardiac content of sphingolipids and glycerophospholipids, major factors contributing to insulin resistance-induced HF [132•]. Although the effects of EMPA in DOXO-induced cardiotoxicity are still under evaluation, preliminary results have shown improved cardiac function in mice treated with EMPA [27]. Of note, EMPA showed a protective effect against DOXO in H9C2 cells and in DOXO-treated mice [27]. From a mechanistic perspective, EMPA has been shown to increase blood ketone levels, as beta hydroxybutyrate (βOHB) which, in turn, improves cell viability and restores mitochondrial dysfunction, ultimately reducing ROS generation and increasing intracellular ATP levels in cardiomyocytes [27].

In conclusion, these observations unravel the possibility of repurposing metabolic drugs to restore autophagy and mitochondrial metabolism to treat or prevent DOXO cardiotoxicity.

## The emerging role of gut microbiota-derived metabolites in DOXO cardiotoxicity

Gut microbiota has been shown to be implicated in several cardiovascular and metabolic diseases, such as atherosclerosis [134], dyslipidemia [135], hypertension [136], chronic kidney disease [137], obesity [138], type I [139] and type II [140] diabetes mellitus, as well as HF [141]. The novel emerging approach of metagenomic has permitted to identify new species of bacteria colonizing human gut that were not able to be cultured in vitro [142] and allowed to compare the gut microbiota composition in patients with HF [141]. It is now well accepted that microbiota-derived metabolites from dietary metabolism influence the pathogenesis of cardiometabolic disorders [143]. These molecules are secreted, degraded, or modified by different metabolic pathways active in intestinal bacteria and can directly or indirectly affect the organism, demonstrating how the gut microbiome can be considered a new and independent endocrine organ in the host [144]. Among the most important metabolites produced by gut microbiota, short chain fatty acids including acetate, propionate, and butyrate have shown an interesting effect on cardiac function in animal models [145]. The cardioprotective effects of butyrate are primarily linked to its epigenetic action since it functions as a potent HDAC inhibitor, and HDAC inhibitors are known to protect the heart from maladaptive hypertrophy and ischemic injuries [146–149]. Furthermore, many studies conducted by Raphaeli and colleagues have elucidated the dual activity of butyrate and its prodrugs which, on the one hand, synergize the antitumoral activity of DOXO in cancer models and, on the other hand, protect the cardiomyocytes against DOXO-induced cardiotoxicity [150–152]. Recently, it has been demonstrated for the first time that in vivo oral administration of FBA, a novel synthetic derivative of butyrate, is able to protect the heart from DOXO-induced cardiotoxicity, preventing mitochondrial dysfunction [153•]. Thus, the use of GUT-microbiota-derived metabolite as nutraceutical may represent a new promising therapeutic approach for DOXO cardiotoxicity.

## Conclusion and future perspectives

The impact of major anticancer treatments on cardiac metabolism has long been ignored and only recently has started to be investigated. The emerging view is that cardiac metabolic alterations may be used not only as early markers of iatrogenic cardiac injury but also as targets for pharmacological interventions aimed at restraining the late-onset and chronic cardiotoxicity associated to the use of anthracyclines. In this scenario, repurposing metabolic drugs for the treatment of cardiotoxicity represents an intriguing approach. The new anti-diabetic drug empagliflozin has proven effective in reducing glucose blood levels and, at the same time, rescuing heart function. However, despite these promising cues, the molecular mechanisms behind the cardioprotective effects of empagliflozin are still mysterious since the putative molecular target of the drug, the sodium-glucose co-transporter-2, is not expressed in cardiomyocytes. Other molecules employed for the treatment of metabolic disorder, such as rosiglitazone, exhibited controversial clinical results [58], thus highlighting the need of further work to clarify these inconsistencies. On the other hand, compelling evidence is available in support of the use of metformin, especially given its dual ability to modulate cardiac metabolism on the one side and to induce cancer cell death in an AMPK-dependent manner on the other side. In perspective, the identification of new and previously undescribed players specifically involved in the metabolic adaptations induced by anthracyclines will pave the way towards the design of new therapeutics that may prevent cardiotoxicity without affecting the antineoplastic proprieties of the drug.
